# The relationship between interleukin-6 and C-reactive protein in patients with benign and malignant prostate disease

**DOI:** 10.1038/sj.bjc.6602211

**Published:** 2004-10-26

**Authors:** P A McArdle, D C McMillan, N Sattar, A M Wallace, M A Underwood

**Affiliations:** 1 University Department of Surgery, Royal Infirmary, Glasgow, UK; 2 University Department of Biochemistry, Royal Infirmary, Glasgow, UK; 3 University Department of Urology, Royal Infirmary, Glasgow, UK

**Keywords:** benign prostatic disease, prostate cancer, interleukin-6, C-reactive protein, Gleason score, total PSA

## Abstract

The relationship between interleukin-6 and C-reactive protein was evaluated in patients with benign (*n*=59) and malignant (*n*=86) prostate disease. The correlation coefficients for patients with benign prostatic disease and prostate cancer were *r*_s_=0.632, *P*<0.001 and *r*_s_=0.663, *P*<0.001, respectively. These results indicate that the relationship between interleukin-6 and C-reactive protein is similar in patients with benign and malignant prostate disease.

The systemic inflammatory response is an obligatory response of the body to infection, surgery or trauma. There is also increasing evidence that the presence of a systemic inflammatory response, as evidenced by increased circulating concentrations of C-reactive protein, is associated with early recurrence and poor survival in a variety of common hormone-independent tumours (Neilsen *et al*, 2000; Scott *et al*, 2002; McMillan *et al*, 2003). There is also some evidence that a similar relationship exists in hormone-dependent tumours (Lewenhaupt *et al*, 1990; Albuquerque *et al*, 1995).

A number of factors appear to mediate the increased production of C-reactive protein. In injury, the pro-inflammatory cytokines, interleukin-1, TNF-alpha and interleukin-6 in particular, have been shown to stimulate the production of C-reactive protein (Gabay and Kushner, 1999). In cancer, the factors which determine circulating concentrations of C-reactive protein are less clear, since studies in cell lines and animal tumour models have demonstrated that a number of factors stimulate C-reactive protein production. For example, it has been shown that leukaemia inhibitory factor and ciliary neurotrophic factor are also capable of inducing an acute-phase protein response from the liver (Baumann *et al*, 1993; Henderson *et al*, 1994; Akiyama *et al*, 1997). There is also evidence that soluble receptor subunits involved in interleukin-6 signal transduction, the soluble interleukin-6 receptor and the soluble gp130 receptor may be important in the regulation of interleukin-6 activity and the acute-phase protein response (Narazaki *et al*, 1993; Jones and Rose-John, 2002). Therefore, if in cancer patients C-reactive protein was stimulated by factors other than interleukin-6, then it might be expected that the relationship between interleukin-6 and C-reactive protein would be less strong than that in patients with benign disease.

The aim of the present study was to examine the relationship between interleukin-6 and C-reactive protein in patients with benign disease (BPH) and in prostate cancer.

## PATIENTS AND METHODS

Consecutive patients undergoing diagnostic transrectal ultrasound-guided (TRUS) biopsy of prostate were included in the study. The indication for TRUS biopsy was either a total PSA concentration greater than 4 ng ml^−1^ and/or an abnormal digital rectal examination.

Prior to biopsy, a blood sample was obtained and the serum stored at −20°C prior to analysis. No patient had a digital rectal examination within a 2-week period prior to sampling. No patient was receiving treatment at the time of blood sampling.

None of the patients had participated in a formal screening programme. All patients underwent a minimum of systematic sextant biopsy. A Gleason score was allocated for each tumour. The Gleason score reflects tumour heterogeneity by combining primary and secondary patterns into a composite score, which has been shown to be an important predictor of disease recurrence and survival (Epstein *et al*, 1993). Gleason scores were compressed as recommended previously (Bostwick *et al*, 2000).

The study was approved by the local Research Ethics Committee. All subjects were informed of the purpose and procedure of the study and all gave written consent.

Total PSA was measured using the Bayer ADVIA Centaur Assay system (Bayer PLC, Bayer House, Newbury, UK). Inter-assay variability was <6% for total PSA.

Interleukin-6 concentrations were measured using a sensitive solid-phase enzyme-linked immunosorbent assay (R&D Systems Europe Ltd, Abingdon, UK). The lower level of detection was 0.2 pg l^−1^ and the intra-assay variability was less than 6% over the sample concentration range.

C-reactive protein concentration was measured using a sensitive double-antibody sandwich ELISA with rabbit anti-human C-reactive protein and peroxidase-conjugated rabbit anti-human C-reactive protein. The assay was linear from 0.1 to 5 mg l^−1^ and logarithmic thereafter. Inter-assay variation was less than 10% over the sample concentrations range.

### Statistics

Data are presented as the median (range), and, where appropriate, differences between control and cancer groups were examined using the Mann–Whitney *U* test. Correlations between two variables were calculated using Spearman's rank correlation test. As the distribution of interleukin-6 and C-reactive protein concentrations was skewed, they were logarithmically transformed to illustrate their relationship in patients with benign prostatic disease and prostate cancer. Analysis was carried out using the statistics package SPSS (SPSS Inc., Chicago, IL, USA).

## RESULTS

A total of 145 patients (59 with benign prostatic disease and 86 with prostate cancer) were included in the study. The characteristics of the patients with benign prostatic disease and prostate cancer are shown in Table 1

**Table 1:** Characteristics of patients with benign prostatic disease and prostate cancer

	**Benign prostatic disease (*n*=59)**	**Prostate cancer (*n*=86)**	***P*-value**
Age (⩽70/>70 years)	45/14	52/34	0.069
Stage (T1–T2/T3–T4/Met)		37/37/12	
Gleason score (2–6, 7, 8–10)		46/24/16	
Total PSA (ng ml^−1^)^a^	7.0 (0.8–39)	14.6 (0.5–146)	<0.001
Interleukin-6 (pg ml^−1^)^a^	2.3 (0.7–12.9)	2.3 (0.8–28.1)	0.526
C-reactive protein (mg l^−1^)^a^	2.0 (0.1–21)	1.8 (0.3–50)	0.891

a Median (range).

. The majority of patients with prostate cancer had localised or locally advanced disease and had a low Gleason score. Compared to patients with benign prostatic disease, the cancer patients had higher circulating concentrations of total PSA (*P*<0.001). There were no significant differences in circulating concentrations of interleukin-6 and C-reactive protein between patients with benign disease and prostate cancer.

**Figure 1:**
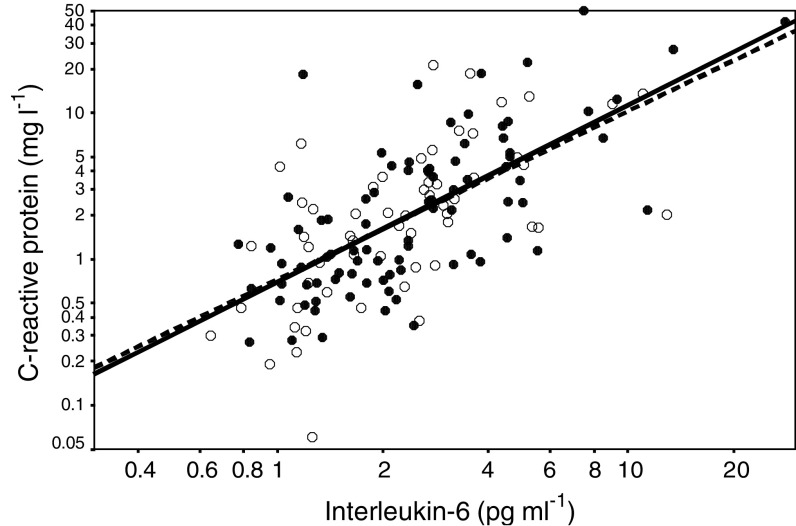
Relationship between circulating concentrations of interleukin-6 and C-reactive protein in patients with benign prostatic disease (○) and prostate cancer (•).

The relationship between circulating concentrations of interleukin-6 and C-reactive protein in patients with benign prostatic disease and prostate cancer is shown in Figure 1

. The correlation coefficients for patients with benign prostatic disease and prostate cancer were *r*_s_=0.632, *P*<0.001 and *r*_s_=0.663, *P*<0.001, respectively.

The relationship between Gleason score, PSA, interleukin-6 and C-reactive protein in the patients with prostate cancer is shown in Table 2

**Table 2:** Relationship between inflammatory parameters and Gleason score in patients with prostate cancer

	**Gleason score 2–6 (*n*=46)**	**Gleason score 7 (*n*=24)**	**Gleason score 8–10 (*n*=16)**	***P*-value**
Age (⩽70/>70 years)	31/15	12/12	9/7	0.343
Stage (T1–T2/T3–T4/Met)	30/14/2	4/17/3	3/6/7	<0.001
Total PSA (ng ml^−1^)^a^	11.0 (0.5–76)	19.8 (3.7–116)	18.7 (4.8–146)	0.025
Interleukin-6 (pg ml^−1^)^a^	1.9 (0.8–11.4)	3.1 (0.8–28.1)	3.0 (1.1–9.3)	<0.005
C-reactive protein (mg l^−1^)^a^	1.1 (0.3–18.7)	2.7 (0.3–50)	3.3 (0.8–12.5)	<0.005

a Median (range).

. With increasing Gleason scores, there were significant increases in total PSA (*P*<0.05), interleukin-6 (*P*<0.01) and C-reactive protein (*P*<0.01). There was a significant correlation between Gleason score and total PSA (*r*_s_=0.364, *P*=0.001), interleukin-6 (*r*_s_=0.311, *P*=0.004) and C-reactive protein (*r*_s_=0.304, *P*=0.004). There was no significant association between total PSA and either interleukin-6 or C-reactive protein.

## DISCUSSION

In the present study, there were no significant differences in the circulating concentrations of either interleukin-6 or C-reactive protein between patients with benign prostatic disease and those with prostate cancer. Furthermore, the relationship between interleukin-6 and C-reactive protein was similar in both patient groups.

In the cancer patients, there was a significant increase in both interleukin-6 and C-reactive protein concentration with increasing tumour grade. These results are also consistent with previous observations that concentrations of interleukin-6 and its receptor are increased in patients with a Gleason score greater than 7 (Shariat *et al*, 2001). This might suggest that the tumour was responsible for the increased production of interleukin-6. However, the increases in interleukin-6 and C-reactive protein were independent of total PSA.

An alternative explanation would be that the source of interleukin-6 is the host inflammatory cells. It is therefore of interest that recent studies have shown that a pronounced inflammatory infiltrate in the tumour was associated with poor outcome in patients with prostate cancer (Irani *et al*, 1999; McArdle *et al*, 2004).

Taken together, these observations confirm that interleukin-6 is predominantly responsible for the elaboration of C-reactive protein. Furthermore, they suggest that interleukin-6 is produced primarily by the host rather than the tumour. Moreover, it may also be that interleukin-6, produced by tumour-infiltrating leucocytes, stimulates tumour cell proliferation and promotes leucocyte recruitment as part of an autocrine growth factor loop (Corcoran and Costello, 2003).

In summary, the results of the present study indicate that the relationship between interleukin-6 and C-reactive protein is similar in patients with benign and malignant prostate disease.
